# Treatment of Gall-Stone, &c.

**Published:** 1889-08

**Authors:** J. A. Comingor

**Affiliations:** Indianapolis, Ind.


### TREATMENT OF GALL-STONE SUCCESSFUL BY MASSAGE OR "PUMPING THE LIVER."

BY J. A. COMINGOR, M. D., INDIANAPOLIS, IND.

As the treatment of gall-stones is now being freely discussed in the medical journals, I have a case in mind that might be of interest to the profession:

The patient, a doctor, 50 years of age, from exposure in November, 1885, was suddenly stricken down with the usual symptoms of obstruction of the gall-duct. Of course, jaundice followed. He was treated eleven weeks before I saw him.

From his attendants I received the history of his case and their treatment. The treatment was so full and complete, that there was nothing I could think of to suggest, in the therapeutic line that they had not used. I therefore suggested, that we apply the principle of massage to the case. This we did, and after we were through with the movements, it reminded me so strikingly of pumping, that I called it "pumping the liver." I proceeded by placing my hands on the ribs over the liver, making firm and quick pressure downwards letting up and repeating, say for five minutes, and then requested that it be repeated two or three times during the night. Up to that time no trace of bile had been discovered in the dejections. The following day a large quantity of dark bilious matter passed from the bowels, soon followed by scores of gall-stones. Improvement set in from that day and continued for at least a fortnight, when from some unknown cause another blockade was on hand. The pumping process was resorted to again with the same desirable result as on the former occasion. After this the patient experienced no further trouble and made a complete recovery.

The principle of the treatment and its application laid down are so simple, and the results in the case above reported were so satisfactory, that the treatment suggested seems worthy of further trial and reports from those who may adopt it.—*Va. Med. Mo.*

